# Giant adrenal myelolipoma, a rare urological issue with increasing incidence: a case report

**DOI:** 10.4076/1757-1626-2-8863

**Published:** 2009-09-01

**Authors:** Stavros I Tyritzis, Ioannis Adamakis, Vasileios Migdalis, Dimitrios Vlachodimitropoulos, Constantinos A Constantinides

**Affiliations:** 1 Department of Urology, Athens University Medical School, “LAIKO” Hospital17 Agiou Thoma str., Athens, 11527Greece; 2 Department of Pathology, Evgenideio Hospital20 Papadiamantopoulou Str, Athens. 11528Greece

## Abstract

**Introduction:**

Adrenal myelolipomas are relatively rare, non-functioning benign tumours composed of mature fatty and active hematopoietic elements. They can be asymptomatic, even if their size is massive. Diagnosis is relatively simple using ultrasound, computed tomography and magnetic resonance imaging. Surgical resection through an extraperitoneal approach is advocated in cases of symptomatic or large myelolipomas exceeding 5-cm in diameter. Their low incidence seems to be increasing from 0.2% to 10% during the last decade.

**Case presentation:**

We present a case of a giant adrenal myelolipoma in a 68-year-old Caucasian male, who was presented with left lumbar pain. Renal ultrasound, CT and MRI demonstrated a well demarcated mass, with a maximum diameter of 10-cm. The differential diagnosis comprised the adrenal myelolipoma, the retroperitoneal liposarcoma and the renal angiomyolipoma. Thus, the patient was subjected to a left adrenalectomy.

**Conclusion:**

Multiple theories have been proposed for the increasing frequency and natural course of the adrenal myelolipoma, with chronic adrenal stimulation and the contemporary stressful lifestyle to be the most appealing. Surgical treatment is advocated through an extraperitoneal approach because of the quicker recovery of the patient and the smaller postoperative complication rate.

## Introduction

Adrenal myelolipoma is an uncommon urological entity, which has an increasing incidence. Despite its benign biology, when presented as a giant tumor, the urologist can face diagnostic dilemmas or even an acute clinical setting. We present a case of an asymptomatic giant adrenal myelolipoma, one of the largest reported in the literature and discuss issues concerning the diagnosis, the management and the proposed theories for the increased prevalence of this lesion.

## Case presentation

**Figure 1.**
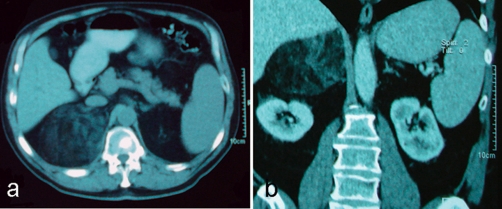
**(a)** Enhanced computed tomography (CT) of the upper abdomen, showing a large heterogenous mass, covering the upper right retroperitoneal space, **(b)** Magnetic resonance imaging (MRI) of the mass, which demonstrates clearly the origin of the mass from the adrenal gland. The right kidney is slightly deviated.

A 68-year-old Caucasian male, with history of untreated left nephrolithiasis, was presented to our hospital, complaining of left lumbar pain, typical of a left renal colic. During the kidney ultrasound, a large, well demarcated mass, with a maximum diameter of 10-cm was demonstrated, in the upper pole of the right kidney, mimicking a renal angiomyolipoma. Contrast-enhanced computed tomography (CT) was performed the next day, verifying a heterogeneous mass with predominant fatty components in the right retroperitoneal space, measuring approximately 12-cm in diameter, slightly compressing the right kidney and vena cava (Figure 1a). Magnetic resonance imaging (MRI) was performed and the findings were classic for myelolipoma (Figure 1b). The tumour was predominantly hyperintense with signal intensity similar to fat on T1/T2-weighted images and lost signal on the fat-suppressed sequences. No other pathological findings were recorded, apart from a large lower pole calculus of the left kidney, with a diameter of 1.4-cm, evidential of the patient’s nephrolithiasis. The differential diagnosis comprised the adrenal myelolipoma, the retroperitoneal liposarcoma and the renal angiomyolipoma.

**Figure 2.**
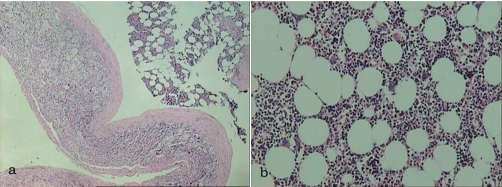
**(a)** Myelolipoma attached to the adrenal gland (H & E stain, ×25), **(b)** Typical hstological features of myelolipoma comprising adipose tissue and poorly differentiated plasma cells with large, hyperchromatic nuclei (H & E stain, ×100).

Surgery was performed through a right subcostal incision for the extraperitoneal approach of the right adrenal gland. The mass was totally dissected from the upper pole of the right kidney and excised en-block with the right adrenal gland. The macroscopic examination of the mass revealed a giant mass measuring 19 × 12 × 2 cm, surrounded by a thin capsule. Yellow adipose tissue was unevenly scattered on the capsule with varying amounts of red hematopoietic tissue (Figure 2). The pathology report showed a giant myelolipoma of the right adrenal gland.

The patient had an uneventful postoperative course and was discharged on postoperative day 4.

## Discussion

Adrenal myelolipomas are also called incidentalomas, since their diagnosis is based on autopsy or imaging techniques performed for reasons unrelated to adrenal diseases. They are relatively rare, non-functioning benign tumors composed of mature fatty and active hematopoietic elements. Their incidence ranges from 0.01% to 0.2% and less than 300 cases were reported until 2000 [1]. Nevertheless, their prevalence seems to be increasing up to 10%, due to novel and enhanced imaging techniques [2]. Is this however the only reason for this increase?

Multiple theories have been proposed for the aetiology and natural course of the adrenal myelolipoma [3-5]. The most widely accepted theory is adrenocortical cell metaplasia in response to stimuli, such as necrosis, inflammation, infection or stress [6]. The chronic adrenal stimulation, as it is demonstrated by the high incidence of the disease to the elderly [7], could generate the development of benign or malignant tumors. Cushing’s disease, hypertension, diabetes and obesity are often related to adrenal myelolipomas and could be characterized as major adrenal stimuli. We could also speculate that the contemporary stressful lifestyle and imbalanced diet could be implicated to the pathogenesis of this tumour. Another unusual and unexplained observation is the predominance of the tumour in the right adrenal gland, as reported in several series [8].

Diagnosis of adrenal myelolipomas can be unproblematic with the use of ultrasound, contrast-enhanced CT, MRI or positron emission tomography (PET). We believe that fine-needle aspiration bears the risk of rupture, especially in cases of giant lesions and spillage when a malignant neoplasia is encountered. No endocrinological evaluation is needed, since myelolipomas are non-functioning tumours.

The management of adrenal myelolipomas should be individualized. A small, less than 5-cm, asymptomatic myelolipoma could be followed-up over a 1-2 year period with imaging controls. On the contrary, a symptomatic lesion or a large >5-cm myelolipoma should be surgically excised, since there are reports of spontaneous rupture and haemorrhage of the mass presented with life-threatening cardiovascular shock [9]. We prefer the extraperitoneal approach rather than a midline incision, because of the quicker recovery of the patient and the smaller postoperative complication rate. The laparoscopic approach seems to be gaining ground on the management of adrenal tumours. On the other hand, this procedure is not indicated for masses larger than 10-cm or including adhesions and infiltration of the surrounding structures [10].

## Conclusion

The adrenal myelolipoma is a rare urological entity, which seems to increase its frequency, probably due to causes affecting primarily the function and physiology of the adrenals. Our case described one of the largest ever reported myelolipomas, which despite its size, can be rather asymptomatic. However, even with the contemporary imaging modalities, precise diagnosis is difficult. Thus, we advocate open surgical management, since a laparoscopic approach might be extremely complicated in cases of such large tumours.

## Abbreviations

CT: computed tomography
MRI: magnetic resonance imaging
PET: positron emission tomography
